# Social Determinants and Oral Health Among Indigenous and Non-Indigenous Older Adults in La Araucanía, Chile: Cross-Sectional Study

**DOI:** 10.2196/85075

**Published:** 2026-09-29

**Authors:** Fernanda Muñoz-Sepúlveda, Karen Danke Hausdorf, Víctor Beltrán Varas, Claudia Acevedo Atala, Leonardo López, Alejandro Herrera

**Affiliations:** 1 Clinical Investigation and Dental Innovation Center (CIDIC), Dental School and Center for Translational Medicine (CEMT-BIOREN) Universidad de La Frontera Temuco Chile; 2 Facultad de Odontología Universidad San Sebastián Santiago Chile; 3 Instituto de Informática Educativa Universidad de La Frontera Temuco Chile; 4 Facultad de Ciencias Agropecuarias y de Medioambiente Universidad de La Frontera Temuco Chile

**Keywords:** delivery of health care, digital health, geriatric dentistry, health inequities, health services accessibility, human rights, Indigenous peoples, rural population

## Abstract

**Background:**

Social disparities in oral health persist, disproportionately affecting Indigenous populations.

**Objective:**

This study aims to compare oral health status, dental care access, treatment needs, and social determinants between Mapuche and non-Mapuche older adults in the La Araucanía Region of Chile, within a hybrid dental care model, and to explore whether ethnicity is associated with these outcomes, guided by the World Health Organization (WHO) Commission on Social Determinants of Health (CSDH) framework.

**Methods:**

We conducted a cross-sectional exploratory analytic study using data from 480 adults aged 60 years or older who received dental care through a hybrid teledentistry and mobile clinic model (Geriatric Dental Specialties Tele-Platform [TEGO]) in 4 municipalities of La Araucanía. The analytic sample was restricted to community-dwelling, functionally independent older adults. Outcomes included oral health status (nonfunctional dentition, edentulism, cavitated caries, and periodontitis), dental care access (fixed prosthetic rehabilitation and unmet denture need), and dental treatment needs (restorative and prosthetic). Structural (sex, age, education, rurality, and ethnicity) and intermediary determinants (behavioral, biological, psychosocial, and health system access) were analyzed following the WHO-CSDH framework. Bivariate analyses compared outcomes between Mapuche and non-Mapuche participants. Multivariable logistic regression models examined associations between determinants and outcomes.

**Results:**

A total of 471 older adults (mean age 72.8, SD 7.7 years) were included; 76.9% (362/471) had nonfunctional dentition, 18% (85/471) were edentulous, 25.6% (98/383) had cavitated caries, and 46% (154/335) had periodontitis. In bivariate analyses, Mapuche participants showed lower educational attainment, higher rural residence, poorer oral health, more behavioral risk factors (eg, infrequent toothbrushing and higher sugar intake), greater restorative needs, and reduced access to complex dental care compared with non-Indigenous peers. In multivariable analyses, female sex was associated with increased odds of edentulism but lower odds of periodontitis. Lower education was associated with nonfunctional dentition (odds ratio [OR] 4.28, 95% CI 2.34-7.88; *P*<.001) and edentulism (OR 3.04, 95% CI 1.52-6.09; *P*=.002). Mapuche ethnicity was associated with higher prevalence of cavitated caries (OR 2.40, 95% CI 1.25-4.63; *P*=.009) and periodontitis (OR 2.43, 95% CI 1.21-4.88; *P*=.01). Dental care access was associated with behavioral factors (brushing, interproximal hygiene, and sugar intake) and structural determinants (age, sex, and rurality), while restorative and prosthetic needs were linked to sugar consumption and tobacco use, respectively.

**Conclusions:**

Oral health inequalities among older adults in La Araucanía were associated with both structural and behavioral determinants. Although Mapuche participants exhibited poorer oral health, higher restorative needs, and reduced access to complex dental care in bivariate analyses, ethnicity remained associated only with cavitated caries and periodontitis in adjusted models. These findings underscore the importance of incorporating social determinants into oral health planning for underserved older adults and highlight the potential utility of data generated through digitally supported territorial care models for informing equity-oriented oral health strategies.

## Introduction

Oral health disparities are a persistent global challenge, disproportionately affecting socially marginalized groups, including older adults and Indigenous populations [1-5]. These inequities are shaped by social determinants of health—the conditions in which people live, work, grow, and age [6]. The World Health Organization (WHO) Commission on Social Determinants of Health (CSDH) framework critically distinguishes between structural determinants (eg, socioeconomic position, education, ethnicity, age, and place of residence) and intermediary determinants (material circumstances, biological factors, psychosocial factors, and health system access), which jointly influence oral health outcomes [6-9]. Among older adults, multiple studies have documented robust social gradients in outcomes such as edentulism and oral health–related quality of life [10-15].

Despite global efforts to improve oral health among older adults, progress has been limited, highlighting the need for approaches that account for contextual complexities and structural inequities impacting health outcomes [16,17]. Indigenous populations worldwide experience some of the most persistent and systemic oral health inequities, deeply rooted in historical and structural injustices [2-5,18]. Older Indigenous populations may face compounded barriers to accessing oral health care due to the intersection of age, ethnicity, socioeconomic status, and geographic location [19].

Chile’s Indigenous population is among those experiencing the highest levels of aging in the region, with a growing proportion of older adults, including a notably high share of people aged 80 years or older [20]. Approximately 8.8% of the national population self-identifies as Indigenous, and about 16% of this group is aged 60 years or older [21]. Nearly 80% of Indigenous people in Chile belong to the Mapuche, who have historically inhabited Wallmapu, the ancestral territory of the Mapuche people spanning present-day Chile and Argentina; its western portion, Ngulu Mapu, corresponds to what is now south-central Chile [21,22]. In recent decades, a considerable proportion of the Mapuche population has migrated to urban and semiurban areas nationwide, a process associated with longstanding structural inequities, loss of ancestral lands, and limited economic opportunities in rural communities [22,23]. Despite their demographic weight and historical relevance, Mapuche communities continue to experience pronounced health inequities compared with their non-Indigenous peers. At age 60, Mapuche individuals have a life expectancy 7.5 years shorter than their non-Indigenous counterparts, along with lower total and disability-free life expectancy and a greater number of years lived with disability [24,25]. These disparities are also reflected in documented differences in metabolic health, including higher levels of insulin resistance, as well as a greater prevalence of mental and communication impairments compared with non-Mapuche older adults [26,27]. Together, these findings illustrate broader inequalities in general health and aging conditions affecting Mapuche older adults.

Oral health status reflects similar social and health gradients observed among Indigenous populations worldwide. Several studies have documented a high prevalence of untreated dental caries, suboptimal oral hygiene practices, and limited access to preventive oral health services among Chilean Indigenous children and adults [28-31], as well as among Indigenous communities globally [2,3,32]. But despite growing awareness of oral health disparities, research on Indigenous populations in Latin America and Chile remains scarce.

In addressing persistent barriers to oral health care among vulnerable populations, innovative service delivery models, such as telehealth and mobile clinics, have gained traction for their ability to overcome geographic isolation and specialist shortages [33,34]. These approaches have shown considerable promise in expanding access to both general and oral health services, alleviating the burden of chronic diseases, and supporting culturally safe, community-centered models of care for Indigenous populations [34-36]. Such strategies are particularly relevant given the strong preference among many older Indigenous individuals to age in place, often in settings characterized by limited financial resources and scarce health infrastructure [37]. At the global level, digital health research targeting older Indigenous populations is gaining momentum—particularly in high-income countries—through the evaluation of innovations such as wearable technologies, videoconferencing, and mobile telemedicine clinics [38-42]. However, these advances remain less common in low- and middle-income countries, where disparities in technological infrastructure, digital literacy, cultural adaptation, and resource availability pose significant challenges to the implementation and sustainability of culturally appropriate telemedicine and eHealth solutions [20,43].

In response to these challenges, a pilot initiative was implemented in a Mapuche community, introducing a hybrid dental care model designed to reduce territorial barriers to oral health services. The model integrates outreach clinical services delivered through a mobile unit with digitally supported coordination and remote specialist consultation, linking primary-level care with higher-level clinical expertise. Hybrid care approaches that combine digital and in-person services have been proposed as strategies to improve access and continuity of care in underserved populations [44]. The digital infrastructure supporting this model was provided by the Geriatric Dental Specialties Tele-Platform (TEGO), a web-based system that integrates electronic health records with structured medical-geriatric and odontogeriatric assessment modules and facilitates interdisciplinary collaboration [29,45-47].

Building on this initial experience, the care model was subsequently expanded to rural and urban municipalities across the La Araucanía Region. Although not exclusively targeted at Indigenous populations, the program engaged both Mapuche and non-Mapuche older adults across diverse settings. This broader implementation generated data that enabled the examination of associations between structural and intermediary social determinants of health and oral health outcomes in these populations.

Accordingly, this study aims to compare oral health status, dental care access, treatment needs, and social determinants of health between Mapuche and non-Mapuche older adults in the La Araucanía Region of Chile receiving care through a hybrid dental care model and to explore the association between ethnicity and these outcomes within the WHO-CSDH framework.

## Methods

### Ethical Considerations

The project was approved by the Ethics Committee of the Universidad de La Frontera (decision 109/22). All participants provided written informed consent, and strict confidentiality protocols were followed, including anonymization prior to analysis.

### Overview

This cross-sectional exploratory analytic study is based on the integrated analysis of data collected at the conclusion of a community-based oral health intervention conducted in the La Araucanía Region of Chile. The intervention corresponded to the regional implementation of the previously described hybrid dental care model and was delivered through a mobile clinical unit serving rural and urban municipalities. Services included geriatric dental assessment and treatment, preventive and restorative care, prosthetic evaluation, and referral management according to treatment complexity. Clinical information, coordination of care, and specialist consultation were supported through the TEGO digital platform following standardized protocols described in previous publications [29,45,47,48].

### Participants and Setting

The La Araucanía Region is distinguished by its high proportion of Indigenous Mapuche population (34.2%) [21]. The region continues to experience persistent socioeconomic disadvantages, including elevated poverty rates, a predominantly rural context, and pronounced health inequities, particularly reflected in limited access to preventive and routine oral health care services [49,50].

In response to these challenges, a community-based oral health intervention program was implemented with funding from a national Presidential Grant (exempt resolution number 472). Initial recruitment was conducted by telephone using databases provided by the regional branch of the National Senior Citizen Service, in collaboration with local community organizations. Municipalities were selected based on logistical feasibility and implementation criteria rather than probabilistic sampling. Program eligibility included adults aged 60 years or older, residing in the region, and physically and cognitively able to attend dental appointments.

Between January and September 2023, a total of 480 older adults received care through this program, which delivered services through scheduled outreach visits using a mobile dental clinic in 4 municipalities: 2 rural (Cholchol and Los Sauces) and 2 urban (Temuco and Angol). The intervention was implemented in coordination with municipalities and community organizations, including the Araucanía Regional Federation of Older Adults, which brings together municipal-level unions of older adult clubs, as well as local community groups such as church organizations.

This study was based on clinical and assessment data collected during program implementation and recorded through the TEGO digital platform. For the present study, eligibility criteria included being a community-dwelling, functionally independent older adult, defined as a Barthel Index score ≥90. Consequently, 9 institutionalized individuals were excluded, resulting in a final sample of 471 participants. Figure 1 presents the detailed participant flow, including eligibility assessment, application of inclusion and exclusion criteria, and the final analytic sample.

**Figure 1:**
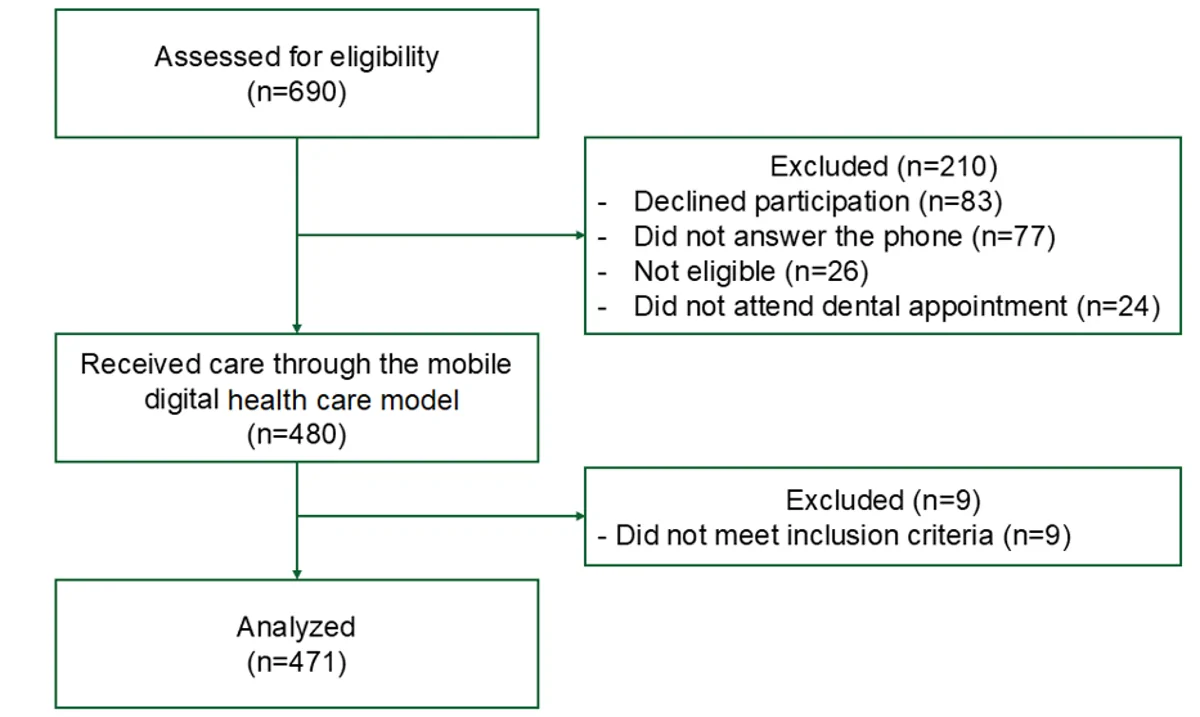
Participant flow diagram.

### Conceptual Framework

The WHO-CSDH framework informed the research question, variable selection and classification, and interpretation of findings by conceptualizing oral health as shaped by processes of social stratification and the unequal distribution of power, resources, and opportunities [6]. This framework distinguishes between structural determinants—such as socioeconomic and political context and axes of social stratification, including education, ethnicity, and place of residence—and intermediary determinants, which encompass material conditions, health-related behaviors, psychosocial factors, and access to health systems. Within this framework, ethnicity was conceptualized as a structural determinant reflecting social position, guiding comparisons of oral health outcomes across groups.

The use of the WHO-CSDH framework is particularly relevant in this study context, as it allows oral health inequalities to be examined beyond individual-level risk factors by situating them within broader social and systemic conditions. This is especially important in Indigenous populations, where health disparities are shaped by historical, social, and territorial inequities that are not fully captured by conventional epidemiological approaches focused on proximal determinants [9]. By organizing variables into structural determinants, intermediary determinants, and oral health outcomes, this framework supports a more comprehensive assessment of how health inequalities are patterned across social groups and how different levels of determinants coexist.

Consistent with the WHO-CSDH model, structural and intermediary determinants are theorized to be hierarchically related through pathways linking social position to health outcomes. However, given the cross-sectional design, the present study examines the distribution of these determinants and their associations with oral health outcomes without inferring causal relationships.

### Data Collection and Variable Structure

Clinical data were collected by 2 final-year dental interns under direct supervision of 2 experienced dentists (CAA and FM-S), using a standardized clinical protocol under uniform examination conditions. Examinations were conducted by the same team that implemented the initial pilot intervention in the Mapuche community, and the regional phase began 1 month later, maintaining the same examiners, instruments, and diagnostic criteria.

Prior to implementation, examiners underwent structured theoretical and supervised clinical training to apply the complete TEGO clinical record, which includes medical-geriatric, odontogeriatric, behavioral, and prosthodontic assessment modules. Training covered caries detection, periodontal examination, prosthetic evaluation, and standardized collection of self-reported variables (eg, oral hygiene practices and service use).

Formal calibration with assessment of interexaminer and intraexaminer reliability was conducted for dental caries using the International Caries Detection and Assessment System (ICDAS). Interexaminer and intraexaminer κ values were 0.66 and 0.78-0.85, respectively [29]. Periodontal and prosthetic assessments were recorded using standardized objective diagnostic criteria under direct supervision to ensure procedural consistency.

All findings were recorded directly into the TEGO [45,47], ensuring standardized data capture and quality control procedures.

Guided by the WHO-CSDH framework, variables were classified as structural determinants, intermediary determinants, or oral health outcomes.

### Structural Determinants

Structural determinants were defined as variables reflecting the broader socioeconomic context that shapes health equity through social stratification. These included: sex (male or female, self-reported); age, recorded as a continuous variable in years and categorized as 60-69 years, 70-79 years, or ≥80 years; and ethnic self-identification, classified as Mapuche (based on self-reported Indigenous affiliation) or non-Mapuche. Educational attainment was dichotomized as no formal or only primary education (≤8 years) vs secondary or higher education (>8 years).

Socioeconomic status was approximated using Chile’s Social Registry of Households (RSH), a national administrative classification used to target social programs. The RSH assigns households to 7 socioeconomic percentiles based on an algorithm that integrates income sources, household composition, and indicators of vulnerability (eg, age, disability, and access to goods and services) [51]. For this study, households were dichotomized into the most vulnerable group (0%-40%) vs higher percentiles (>40%).

Additional structural variables included place of residence, classified as urban or rural according to municipality-level administrative definitions [52], and health insurance coverage, categorized according to the Chilean health system as the National Health Fund (FONASA), the health systems for the armed forces and police (National Defense Social Security Fund [CAPREDENA] and Carabineros de Chile Social Security Directorate [DIPRECA]), the Comprehensive Health Care and Reparation Program for Victims of Human Rights Violations (PRAIS), private health insurance institutions (ISAPREs), or no health coverage.

### Intermediary Determinants

#### Overview

These variables represent the mechanisms through which structural determinants influence health, including behavioral, biological, and psychosocial factors and access to care.

#### Behavioral Factors

Included oral hygiene practices, dietary habits, and smoking behavior, which are known to influence oral and general health [53-56]. Participants reported the frequency of tooth or denture brushing, interproximal hygiene (yes or no), and use of toothpaste (yes or no). Cariogenic dietary patterns were assessed through questions about the consumption of sugary foods and beverages between meals—such as candies, cookies, cakes, soda, sweetened juices, or sugar-added drinks (eg, coffee, tea, mate, or milk; yes or no). Daily water intake was also recorded and categorized as less than or more than 2 liters. Smoking status was documented through a yes or no question regarding current tobacco use.

#### Biological Factors

Several biological conditions—multimorbidity, polypharmacy, dry mouth, and swallowing disorders—were included as biological factors that may influence oral health, given their established association with impaired oral function, increased risk of caries, periodontal disease, and challenges in maintaining oral hygiene in older adults [57-60]. Multimorbidity and polypharmacy were obtained through self-report. Multimorbidity was defined as the presence of 2 or more chronic conditions, as reported by participants [61], while polypharmacy was defined as the regular use of 5 or more medications per day [62], based on participants’ declarations of medication intake. The subjective sensation of dry mouth was recorded, and swallowing disorders were assessed using the Eating Assessment Tool-10 (EAT-10), a validated screening instrument; scores ≥3 indicate a positive screen for swallowing difficulties [63,64].

#### Psychosocial Factors

Psychosocial factors included variables related to social relationships, used as proxies for social isolation—a recognized risk factor for health in older adults [11]. Living arrangements were recorded and categorized as living alone or living with others. Perceived social support was assessed based on participants’ subjective perception of receiving emotional or practical help when needed (yes or no).

#### Health System Access

Access to oral health services was assessed through various direct and indirect indicators. Self-reported time since last dental visit, categorized as ≤2 years or >2 years, was used as a direct indicator of access. The presence of fixed dental rehabilitation (eg, crowns and fixed bridges) and the presence of dental implants were used as indirect indicators of a history of better access to specialized dental treatment, whereas unmet denture need (absence of a removable dental prosthesis in individuals who were clinically identified as requiring a partial or complete denture) was used as a proxy for barriers to care.

In Chile, treatments such as restorations and removable dentures are commonly provided within primary care, although prosthetic programs do not cover all individuals [65]. In contrast, more advanced treatments, including dental implants and fixed prostheses, are typically available only through secondary care or private services. These indicators therefore reflect not only the availability of services but also the types of services they accessed, going beyond the traditional measure of time since the last dental visit. This approach aligns with previous studies that have used restorative, prosthetic, and visit-related measures as proxies for access to care and oral health inequalities [66,67].

#### Oral Health Status

Oral health status was assessed through clinical examination and included the number of natural teeth, functional dentition, edentulism, cavitated caries, and periodontal status. The number of natural teeth was recorded as the total count of teeth remaining in the oral cavity, while nonfunctional dentition was defined as the presence of fewer than 20 teeth, regardless of their location. Edentulism was defined as the complete absence of natural teeth.

Cavitated caries were defined as lesions corresponding to the decayed (D) component of the decayed, missing, and filled teeth (DMFT) index, operationalized as ICDAS codes 4 to 6.

Periodontal status was assessed through a full-mouth periodontal examination, recording plaque levels, probing depth (PD), clinical attachment level (CAL), bleeding on probing, suppuration, and gingival recession at 6 sites per tooth, as well as furcation lesions and tooth mobility [68]. Based on these measures, participants were classified as having gingival health (<10% bleeding sites with PD ≤3 mm [69]), gingivitis (≥10% bleeding sites with PD ≤3 mm), or periodontitis (CAL ≥2 mm on nonadjacent teeth, or buccal/oral CAL ≥3 mm with PD >3 mm on ≥2 teeth [70]). For analytical purposes, periodontal status was dichotomized as presence vs absence of periodontitis.

#### Treatment Needs

Treatment needs were assessed through clinical examination and included both restorative and prosthetic requirements. Restorative treatment need was defined as the number of teeth requiring intervention due to caries, defective restorations, or other conditions requiring restorative care (eg, fractures or noncarious lesions). Including this measure provides a broader estimate of treatment requirements and supports planning and resource allocation.

Normative prosthetic treatment need was operationalized as a binary variable (presence or absence). Participants were classified as having prosthetic treatment need when clinical examination identified complete edentulism without prosthetic rehabilitation, partial tooth loss requiring prosthetic replacement, or existing removable prostheses in inadequate condition requiring repair or replacement. For participants with removable prostheses (complete or partial), functional adequacy was assessed based on stability, retention, and structural integrity. Stability and retention were evaluated following the Kapur index and the prosthodontic evaluation methods from phase 1 of the Third National Health and Nutrition Examination Survey (NHANES III) [71,72], assessing denture movement under manual lateral and vertical forces. Structural integrity was determined by identifying fractures, missing or chipped teeth, and broken clasps or framework components. Prostheses were classified as requiring repair or not requiring repair according to these criteria.

### Statistical Analysis

Descriptive analyses were conducted to characterize the study population. Categorical variables were summarized as frequencies and percentages, and continuous variables as means with SDs or 95% CIs, as appropriate. Bivariate analyses compared structural determinants, intermediary determinants, and oral health indicators by ethnicity (Mapuche vs non-Mapuche). Associations between categorical variables were assessed using the Pearson chi-square test, with *P* values of <.05 considered statistically significant.

Guided by the WHO-CSDH framework, bivariate and multivariable logistic regression analyses were then conducted to examine the associations between structural and intermediary determinants and selected outcomes. Three groups of outcomes were evaluated: clinical oral health outcomes (edentulism, nonfunctional dentition, periodontitis, and caries), access-related outcomes (time since the last dental visit, presence of fixed dental rehabilitation, and clinically unmet denture need), and treatment needs (restorative and prosthetic). Separate multivariable logistic regression models were fitted for each binary outcome (9 models in total) to estimate adjusted associations between structural and intermediary determinants and the outcomes.

Missing data were present in a small proportion of observations for several variables (<10%), and complete-case analysis was therefore considered appropriate given the overall low level of missingness. The proportion of missing data for periodontitis was higher (51/386, 13%). For this outcome, missingness was not significantly associated with most evaluated covariates (all *P*>.05), although excluded participants were older, suggesting a potential age-related component. For all other variables, no substantial differences were observed between included and excluded participants across the evaluated sociodemographic characteristics. Missing periodontal data most likely reflects incomplete clinical examinations rather than item nonresponse, as periodontal assessment requires full-mouth probing in dentate participants. Such incomplete examinations may occur due to participant refusal, logistical constraints during fieldwork, or medical conditions preventing the assessment.

Because some outcomes were only applicable to specific subgroups (eg, dentate individuals or denture users) and some covariates had incomplete observations, the number of observations varied across models; therefore, logistic regression analyses were conducted using complete-case data for each model. Covariates were selected a priori based on the conceptual framework of social determinants of health, rather than through data-driven selection procedures. Indicators of health system access were conceptualized as intermediary determinants; however, in specific models they were also analyzed as outcomes to explore inequalities in access to care. In models examining oral health status outcomes, time since the last dental visit was included as a covariate to account for differential exposure to dental care. Models were also adjusted for the number of natural teeth present, when applicable, given its potential role as a confounder. Results are presented as odds ratios (ORs) with 95% CI, and all analyses were conducted using Stata (version 18; StataCorp LLC).

## Results

### Sociodemographic Characteristics and Structural Determinants

A total of 471 community-dwelling older adults were included in the analysis (Figure 1). Of these, 24.2% (114/471) self-identified as Mapuche. The mean age of the sample was 72.8 (SD 7.7) years, with Mapuche participants being significantly younger than non-Mapuche counterparts (*P*=.006).

Univariate and bivariate descriptive statistics for the study variables are presented in Table 1. Overall, 59.9% (282/471) of participants were female, with no significant differences between Mapuche and non-Mapuche participants (*P*=.62). A total of 126 participants were not registered in RSH, as enrollment in this administrative system is voluntary in Chile. Among those registered (n=345 of 471), most (301/345, 87.2%) were classified within the ≤40% bracket, indicating high socioeconomic vulnerability. No significant differences were observed between Mapuche and non-Mapuche participants (*P*=.10). Regarding health coverage, most participants (405/471, 85.9%) were enrolled in FONASA, 9.8% (46/471) had no health insurance, and 4.2% (20/471) were covered by other schemes (ISAPRE, CAPREDENA/DIPRECA, and PRAIS). Coverage differed by ethnicity; nearly all Mapuche participants (111/114, 97.4%) had FONASA, while non-Mapuche participants showed more variation, including a higher proportion without insurance (45/357, 12.6%; *χ*^2^_2_=16.6; *P*<.001). Compared with their non-Indigenous counterparts, Mapuche individuals had lower educational attainment and resided predominantly in rural municipalities.

**Table 1:** Distribution of structural and intermediary determinants and oral health indicators according to ethnic group.

Variable and category						Total, n (%)			Non-Mapuche, n (%)			Mapuche, n (%)			*P* value	
Structural determinants																
	Sex															.62
		Male			189 (40.1)			141 (39.5)			48 (42.1)					
		Female			282 (59.9)			216 (60.4)			66 (57.9)					
	Age group (years)															.003
		60-69			184 (39.1)			124 (34.7)			60 (52.6)					
		70-79			184 (39.1)			148 (41.5)			36 (31.6)					
		≥80			103 (21.8)			85 (23.8)			18 (15.8)					
	Educational attainment (years completed)															.003
		≤8			252 (53.5)			177 (49.6)			75 (65.8)					
		>8			219 (46.5)			180 (50.4)			39 (34.2)					
	Rurality															<.001
		Rural			188 (39.9)			99 (27.7)			89 (78.1)					
		Urban			283 (60.1)			258 (72.3)			25 (21.9)					
Intermediary determinants																
	Behavioral factors															
		Brushing frequency (times per day; missing=12)													.001	
			<2	67 (14.6)			40 (11.5)			27 (24.1)						
			≥2	392 (85.4)			307 (88.5)			85 (75.9)						
		Interproximal oral hygiene^a^													.38	
			Yes	121 (31.3)			95 (32.5)			26 (27.7)						
			No	265 (68.7)			197 (67.5)			68 (72.3)						
		Sugary snacks consumption													<.001	
			Yes	240 (51)			161 (45.1)			79 (69.3)						
			No	231(49)			196 (54.9)			35 (30.7)						
		Daily water intake (≥2 liters)													.79	
			Yes	170 (36.1)			130 (36.4)			40 (35.1)						
			No	301 (63.9)			227 (63.6)			74 (64.9)						
		Smoking habit													.59	
			Smoker	43 (9.1)			34 (9.5)			9 (7.9)						
			Nonsmoker	428 (90.9)			323 (90.5)			105 (92.1)						
	Biological factors															
		Polypharmacy (missing=38)													.15	
			Yes	72 (16.6)			59 (18.1)			13 (12.2)						
			No	361 (83.4)			267 (81.9)			94 (87.8)						
		Multimorbidity													.35	
			Yes	286 (60.7)			221 (61.9)			65 (57)						
			No	185 (39.3)			136 (38.1)			49 (43)						
		Dry mouth													.06	
			Yes	246 (52.2)			178 (49.9)			68 (59.7)						
			No	225 (47.8)			179 (50.1)			46 (40.3)						
		Swallowing disorders (EAT-10^b^)													.007	
			Yes	38 (8.1)			22 (6.2)			16 (14)						
			No	433 (91.9)			335 (93.8)			98 (86)						
	Psychosocial factors															
		Living arrangement													.008	
			Living alone	142 (30.2)			119 (33.3)			23 (20.2)						
			Living with family	329 (69.8)			238 (66.7)			91 (79.8)						
		Social support													.18	
			Yes	399 (84.7)			298 (83.5)			101 (88.6)						
			No	72 (15.3)			59 (16.5)			13 (11.4)						
	Health system factors															
		Self-reported time since last dental visit (years; missing=4)													.16	
			≤2	229 (49)			180 (50.8)			49 (43.4)						
			>2	238 (51)			174 (49.2)			64 (56.6)						
		Unmet denture need													.44	
			Yes	70 (18.6)			55 (19.5)			15 (16)						
			No	306 (81.4)			227 (80.5)			79 (84)						
		Fixed rehabilitation													.003	
			Yes	75 (15.9)			67 (18.8)			8 (7)						
			No	396 (84.1)			290 (81.2)			106 (93)						
		Dental implants													.16	
			Yes	6 (1.3)			6 (1.7)			0 (0)						
			No	465 (98.7)			351(98.3)			114 (100)						
Oral health status																
	Nonfunctional dentition															.10
		Yes			362 (76.9)			268 (75.1)			94 (82.5)					
		No			109 (23.1)			89 (24.9)			20 (17.5)					
	Periodontitis (missing=51)^a^															<.001
		Yes			154 (46.0)			97 (38.6)			57 (67.9)					
		No			181(54.0)			154 (61.3)			27 (32.1)					
	Cavitated caries (missing=3)^a^															<.001
		Yes			98 (25.6)			60 (20.7)			38 (40.9)					
		No			285(74.4)			230(79.3)			55 (59.1)					
	Complete edentulism (total tooth loss)															.87
		Yes			85 (18)			65 (18.2)			20 (17.5)					
		No			386 (82)			292 (81.8)			94 (82.5)					
Dental treatment need																
	Restorative treatment needs (missing=3)^a^															.001
		No need (0)			177(46.2)			143 (49.3)			34 (36.6)					
		Moderate need (1-3)			174 (45.4)			131 (45.2)			43 (46.2)					
		Severe need (≥4)			32 (8.4)			16 (5.5)			16 (17.2)					
	Prosthetic treatment needs (missing=1)															.13
		Yes			314 (66.8)			232 (64.9)			82 (72.6)					
		No			156 (33.2)			125 (35.1)			31 (27.4)					

^a^Only dentate participants were considered (n=386).

^b^EAT-10: Eating Assessment Tool-10.

### Intermediary Determinants

#### Behavioral Factors

Regarding behavioral determinants, overall, 85.4% (392/459) of participants reported brushing their teeth or dentures at least twice daily, whereas only 3.4% (16/459) reported never brushing. Among participants who were not completely edentulous, 31.9% (123/386) reported performing interproximal dental hygiene. Cariogenic dietary patterns were common, with 51% (240/471) consuming sugary foods or drinks between meals, with an average frequency of 2.3 (SD 0.9) times per day. Mapuche participants reported lower toothbrushing frequency and higher sugary consumption between meals (*P*<.001). Overall, 9.1% (43/471) of participants were current smokers. No statistically significant differences were observed between Mapuche and non-Mapuche participants in smoking status (*P*=.59) or daily water consumption (*P*=.79; Table 1).

#### Biological Factors

In terms of biological factors, the mean number of prescription medications per day was 2.63 (95% CI 2.43-2.83), and 16.6% (72/433) of participants met the criteria for polypharmacy. The mean number of chronic conditions was 2.36 (95% CI 2.20-2.51), with 60.7% (286/471) experiencing multimorbidity. Dry mouth was self-reported by 52.2% (246/471) of participants, while 8.1% (38/471) screened positive for swallowing disorders. Only swallowing disorders showed significant differences by ethnicity, being more prevalent among Mapuche participants (*P*=.007; Table 1).

#### Psychosocial Factors

Overall, perceived social support was high, with 84.7% (399/471) reporting access to emotional or practical help when needed, and 69.8% (329/471) living with family members. Mapuche participants were less likely to live alone compared with non-Mapuche participants (*P*=.008).

#### Health System Factors

Approximately half of participants (229/467, 49%) reported visiting a dentist within the last 2 years, with no significant differences by ethnicity (Table 1). Overall, 18.6% (70/376) of participants had unmet denture need, with no significant differences by ethnicity (*P*=.44).

More complex rehabilitations were less common. Fixed prosthetic rehabilitation was observed only in 15.9% (75/471) of participants (mean fixed rehabilitation per recipient: 3.63, 95% CI 2.88-4.38), while the presence of dental implants was rare (6/471, 1.3%), with a mean of 2.5 (95% CI 0.77-4.22) implants per recipient. Some participants received both types of treatments.

In the bivariate comparisons by ethnicity, no differences were observed in time since the last dental visit. However, Mapuche participants had a significantly lower prevalence of fixed prostheses (*P*=.003). A lower prevalence of implants was also observed among Mapuche participants, although this difference was not statistically significant (Table 1).

#### Oral Health Status

Only 4% (19/471) of participants had 28 or more natural teeth. The mean number of natural teeth present was 12.93 (95% CI 12.13-13.72), with no significant differences between Mapuche and non-Mapuche participants (*P*=.86). Nonfunctional dentition was present in 76.9% (362/471) of participants, while 18.1% (85/471) were edentulous; no significant differences were observed by ethnicity (Table 1).

All participants had a history of dental caries. The mean DMFT index was 22.82 (95% CI 22.23-23.42), ranging from 6 to 32. Among participants aged 65 to 74 years, the mean DMFT was 22.03 (95% CI 21.10-22.95). No significant differences were observed between Mapuche participants (mean DMFT 23.12, SD 5.4) and non-Mapuche participants (mean DMFT 21.6, SD 6.5) within this age group (*P*=.14) or overall (*P*=.09). Tooth loss represented the largest component of the index, accounting for 83.6% (mean 19.07), followed by restorations, which accounted for 15% (mean 3.42). Approximately one-quarter of dentate participants (98/383, 25.6%) had cavitated caries, with a significantly higher prevalence among Mapuche participants (*P*<.001).

Among dentate participants, 20.6% (69/335) had periodontal health, 33.4% (112/335) had gingivitis, and 45.9% (154/335) had periodontitis. Significant differences were observed between Mapuche and non-Mapuche participants (*P*<.001; Table 1).

#### Dental Treatment Needs

Restorative treatment needs were observed in 53.8% (206/383) of participants, and among dentate individuals, the mean number of teeth requiring restorative treatment was 1.25 (95% CI 1.23-2.24). Most participants used dentures (306/470, 65.1%). However, denture repair needs were high: 79.7% (244/306) of denture users required repair or replacement due to poor fit, retention, or structural defects. Overall, normative prosthetic treatment need was 66.8% (314/470). Mapuche participants had significantly greater restorative treatment needs, but prosthetic treatment need did not differ significantly between Mapuche and non-Mapuche participants (Table 1).

### Association of Ethnicity and Other Social Determinants With Oral Health Status

After conducting bivariate analyses for all selected outcomes and covariates, multivariable logistic regression models were fitted to estimate the adjusted effect of each independent variable. Statistically significant bivariate associations are reported in the corresponding table. Given the exploratory nature of the analysis, all predictor variables were included in the multivariable models regardless of their bivariate significance in order to control for potential confounding and obtain adjusted estimates.

In the multivariable logistic regression models, structural determinants exhibited the most consistent associations with oral health outcomes. Age and educational attainment were significantly associated with tooth loss: participants aged 80 years or older had higher odds of nonfunctional dentition (OR 5.55, 95% CI 2.34-13.08; *P*<.001) and complete edentulism (OR 3.75, 95% CI 1.62-8.68; *P*=.002) compared with those aged 60 to 69 years, while ≤8 years of education was linked to increased odds of nonfunctional dentition (OR 4.28, 95% CI 2.34-7.88; *P*<.001) and complete edentulism (OR 3.04, 95% CI 1.52-6.09; *P*=.002). Women had higher odds of edentulism (OR 2.38, 95% CI 1.24-4.58; *P*=.009) but lower odds of periodontitis (OR 0.53, 95% CI 0.31-0.90; *P*=.02). Mapuche ethnicity was associated with higher odds of cavitated caries (OR 2.40, 95% CI 1.25-4.63; *P*=.009) and periodontitis (OR 2.43, 95% CI 1.21-4.88; *P*=.01). Rurality showed no significant associations (Table 2).

**Table 2:** Adjusted associations between structural and intermediary determinants and oral health status outcomes from multivariable logistic regression models. For each binary outcome, a separate multivariable logistic regression model was fitted, including all structural and intermediary predictors. Categorical predictors with more than 2 levels were entered using dummy coding, and the number of natural teeth was included as a covariate.

Variable and category				Nonfunctional dentition		Complete edentulism			Cavitated caries			Periodontitis	
				aOR^a^ (95% CI)	*P* value	aOR (95% CI)	*P* value	aOR (95% CI)		*P* value	aOR (95% CI)		*P* value
Structural determinants													
	Sex^b-d^												
		Male		1.00 (ref)	—^e^	1.00 (ref)	—	1.00 (ref)		—	1.00 (ref)		—
		Female		1.32 (0.76-2.30)	.32	2.38 (1.24-4.58)	.009	0.81 (0.47-1.39)		.44	0.53 (0.31-0.90)		.02
	Age groups (years)^c,d,f^												
		60-69		1.00 (ref)	—	1.00 (ref)	—	1.00 (ref)		—	1.00 (ref)		—
		70-79		2.01 (1.12-3.60)	.02	1.87 (0.89-3.92)	.09	0.58 (0.32-1.05)		.07	1.06 (0.59-1.91)		.84
		≥80		5.55 (2.36-13.08)	<.001	3.75 (1.62-8.68)	.002	0.47 (0.21-1.09)		.07	1.49 (0.69-3.24)		.30
	Educational attainment (years)^b,c^												
		>8		1.00 (ref)	—	1.00 (ref)	—	1.00 (ref)		—	1.00 (ref)		—
		≤8		4.28 (2.33-7.88)	<.001	3.04 (1.52-6.09)	.002	1.61 (0.88-2.95)		.12	0.76 (0.44-1.33)		.34
	Rurality^b-d,f^												
		Urban		1.00 (ref)	—	1.00 (ref)	—	1.00 (ref)		—	1.00 (ref)		—
		Rural		1.52 (0.78-2.97)	.22	1.59 (0.83-3.04)	.15	1.00(0.53-1.89)		.98	1.69 (0.91-3.13)		.09
	Ethnicity^f^												
		None		1.00 (ref)	—	1.00 (ref)	—	1.00 (ref)		—	1.00 (ref)		—
		Mapuche		1.30 (0.62-2.71)	.48	0.85 (0.40-1.82)	.68	2.40 (1.25-4.63)		.009	2.43 (1.21-4.88)		.01
Intermediary determinants													
	Behavioral factors												
		Brushing frequency (times per day)											
			0	1.00 (ref)	—	1.00 (ref)	—	1.00 (ref)		—	1.00 (ref)		—
			≥2	0.8 (0.34-1.89)	.62	0.99 (0.43-2.26)	.98	1.19 (0.55-2.57)		.65	1.16 (0.55-2.48)		.69
		Interproximal oral hygiene^b,c,f^											
			Yes	1.00 (ref)	—	1.00 (ref)	—	1.00 (ref)		—	1.00 (ref)		—
			No	0.37 (0.21-0.65)	.001	0.97 (0.02-0.42)	.002	0.64 (0.33-1.22)		.17	0.8 (0.44-1.45)		.46
		Sugary snacks consumption^d^											
			No	1.00 (ref)	—	1.00 (ref)	—	1.00 (ref)		—	1.00 (ref)		—
			Yes	0.91 (0.52-1.58)	.72	0.67 (0.36-1.22)	.18	1.14 (0.67-1.96)		.62	1.86 (1.11-3.12)		.01
		Daily water intake (≥2 liters)											
			Yes	1.00 (ref)	—	1.00 (ref)	—	1.00 (ref)		—	1.00 (ref)		—
			No	0.99 (0.56-1.74)	.96	1.71 (0.91-3.21)	.09	1.08 (0.62-1.90)		.78	0.9 (0.52-1.56)		.70
		Smoking habit^f^											
			Nonsmoker	1.00 (ref)	—	1.00 (ref)	—	1.00 (ref)		—	1.00 (ref)		—
			Smoker	1.33 (0.53-3.36)	.54	2.31 (0.83-6.39)	.10	1.96 (0.86-4.51)		.11	1.25 (0.49-3.14)		.63
	Biological factors												
		Polypharmacy											
			No	1.00 (ref)	—	1.00 (ref)	—	1.00 (ref)		—	1.00 (ref)		—
			Yes	0.65 (0.31-1.38)	.26	1.28 (0.59-2.78)	.52	0.93 (0.42-2.09)		.86	0.8 (0.38-1.67)		.55
		Multimorbidity^f^											
			No	1.00 (ref)	—	1.00 (ref)	—	1.00 (ref)		—	1.00 (ref)		—
			Yes	0.77 (0.42-1.41)	.40	1.17 (0.59-2.30)	.65	0.59(0.34-1.06)		.07	0.74 (0.42-1.30)		.30
		Dry mouth^c^											
			No	1.00 (ref)	—	1.00 (ref)	—	1.00 (ref)		—	1.00 (ref)		—
			Yes	2.08 (1.19-3.63)	.009	1.7 (0.93-3.10)	.08	1.78 (1.02-3.13)		.04	1.2 (0.71-2.03)		.49
		Swallowing disorders											
			No	1.00 (ref)	—	1.00 (ref)	—	1.00 (ref)		—	1.00 (ref)		—
			Yes	0.56 (0.22-1.43)	.22	0.93 (0.34-2.52)	.88	1.38 (0.58-3.28)		.47	1.05 (0.41-2.67)		.92
	Psychosocial factors												
		Living arrangement											
			With family	1.00 (ref)	—	1.00 (ref)	—	1.00 (ref)		—	1.00 (ref)		—
			Alone	1.19 (0.65-2.18)	.58	1.28 (0.68-2.43)	.44	1.18 (0.64-2.17)		.59	0.83 (0.47-1.46)		.51
		Social support											
			Yes	1.00 (ref)	—	1.00 (ref)	—	1.00 (ref)		—	1.00 (ref)		—
			No	0.51 (0.21-1.24)	.13	2.1 (0.79-5.59)	.13	1.3 (0.56-3.02)		.54	0.73 (0.35-1.55)		.41
	Health System factors												
		Self-reported time since last dental visit (years)^b,c^											
			>2	1.00 (ref)	—	1.00 (ref)	—	1.00 (ref)		—	1.00 (ref)		—
			≤2	1.25 (0.73-2.15)	.41	0.53 (0.28-0.99)	.048	0.75 (0.44-1.28)		.29	1.58 (0.94-2.66)		.08

^a^aOR: adjusted odds ratio.

^b^Indicates variables showing significant bivariate associations with edentulism.

^c^Indicates variables showing significant bivariate associations with nonfunctional dentition.

^d^Indicates variables showing significant bivariate associations with periodontitis.

^e^Not applicable.

^f^Indicates variables showing significant bivariate associations with caries.

Among intermediary determinants, behavioral factors appeared to be the most relevant. Specifically, lack of interproximal hygiene was linked to tooth loss outcomes, while consumption of sugary snacks was associated with increased odds of periodontitis (OR 1.86, 95% CI 1.11-3.12; *P*=.01; Table 2). Psychosocial factors showed no significant associations, and biological factors had limited associations, with dry mouth linked to nonfunctional dentition (OR 2.08, 95% CI 1.20-3.63; *P*=.009) and cavitated caries (OR 1.78, 95% CI 1.02-3.13; *P*=.04). Regarding dental care access, having received care in the past 2 years was associated with lower odds of total edentulism (OR 0.53, 95% CI 0.28-0.99; *P*=.048). These multivariable results, including the 4 oral health outcomes, are summarized in Figure 2.

**Figure 2:**
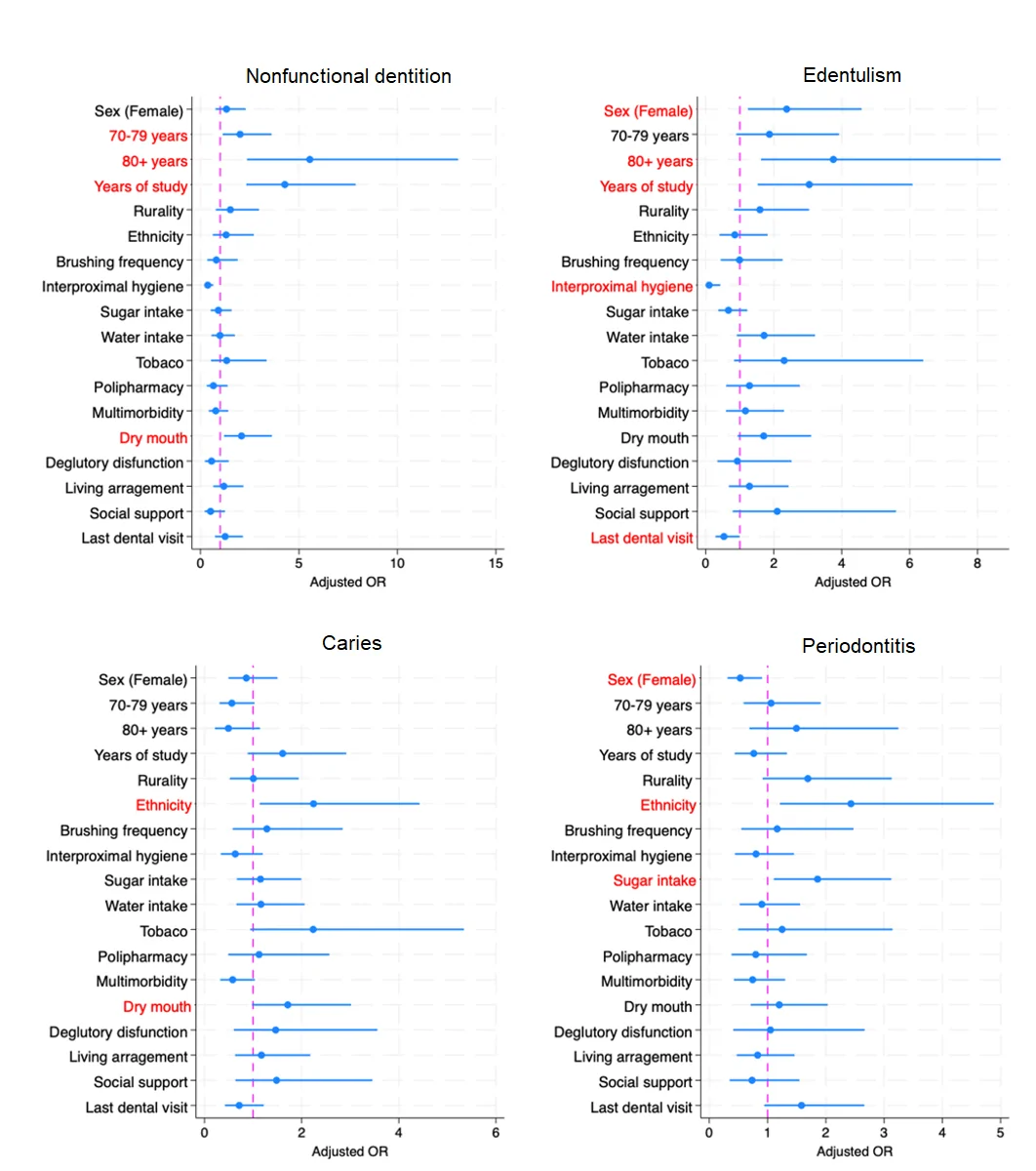
Adjusted associations between predictor variables and oral health status outcomes. Forest plot showing adjusted odds ratios (aORs) and 95% CIs from separate multivariable logistic regression models for nonfunctional dentition, edentulism, cavitated caries, and periodontitis. Points represent aORs, and horizontal bars represent 95% CIs. The vertical line indicates an odds ratio (OR) of 1 (no association). Statistically significant associations are highlighted in red.

### Association of Ethnicity and Other Social Determinants With Access to Dental Care

In the multivariable analysis, variables related to dental care were examined (full results are provided in Multimedia Appendix 1). Time since the last dental visit was associated with toothbrushing frequency (OR 1.92, 95% CI 1.02-3.62; *P*=.04) and interproximal hygiene practices (OR 1.90, 95% CI 1.13-3.19; *P*=.01). Rural residence was associated with higher odds of unmet denture need (OR 2.86, 95% CI 1.31-6.24; *P*=.008), whereas brushing frequency (OR 0.25, 95% CI 0.11-0.57; *P*<.001) was associated with lower odds.

In the adjusted model, women were more likely to have received fixed prostheses (OR 3.46, 95% CI 1.51-7.94; *P*=.003), as were participants aged 80 years or older (OR 5.05, 95% CI 1.68-15.17; *P*=.004). Conversely, participants with ≤8 years of education were less likely to have received fixed prostheses (OR 0.37, 95% CI 0.15-0.91; *P*=.03). Consumption of sugary snacks was also associated with higher odds of having received fixed prosthetic rehabilitation (OR 3.48, 95% CI 1.56-7.78; *P*=.002). Other determinants, including ethnicity, did not show significant associations with this outcome (Figure 3).

**Figure 3:**
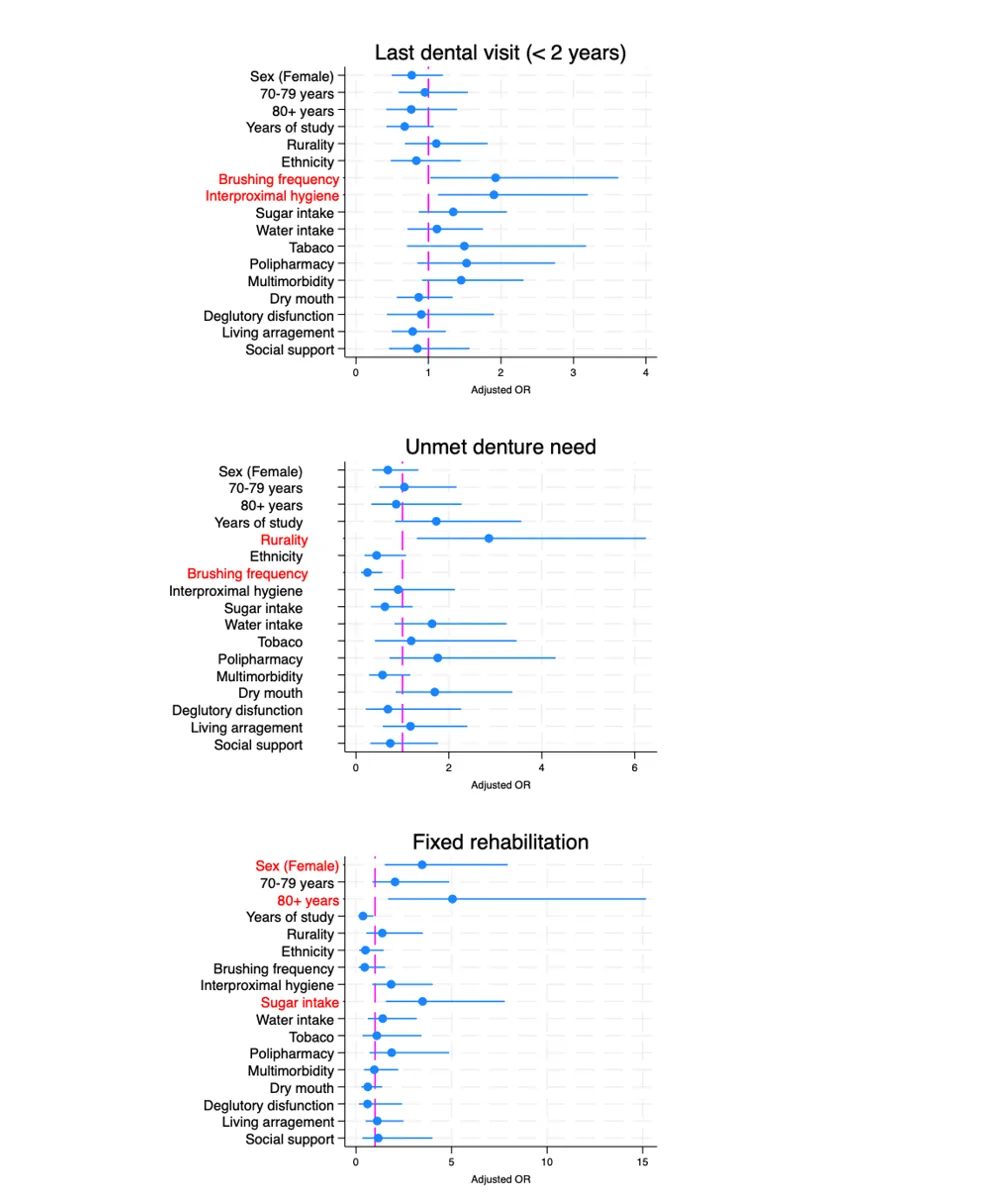
Adjusted associations between predictor variables and access to dental care outcomes. Forest plot showing adjusted odds ratios (aORs) and 95% CIs from separate multivariable logistic regression models for last dental visit, unmet denture need, and fixed rehabilitation. Points represent aORs, and horizontal bars represent 95% CIs. The vertical line indicates an odds ratio (OR) of 1 (no association). Statistically significant associations are highlighted in red.

### Association of Ethnicity and Other Social Determinants With Treatment Needs

When treatment needs were analyzed, no structural determinants were significantly associated with these outcomes. However, higher consumption of sugary snacks between meals was linked to increased odds of requiring restorative treatment (OR 1.69, 95% CI 1.03-2.77; *P*=.03). In addition, current smoking was associated with higher odds of normative prosthetic treatment need (OR 3.49, 95% CI 1.3-9.31; *P*=.01; Figure 4). Full adjusted model results are presented in Multimedia Appendix 2.

**Figure 4:**
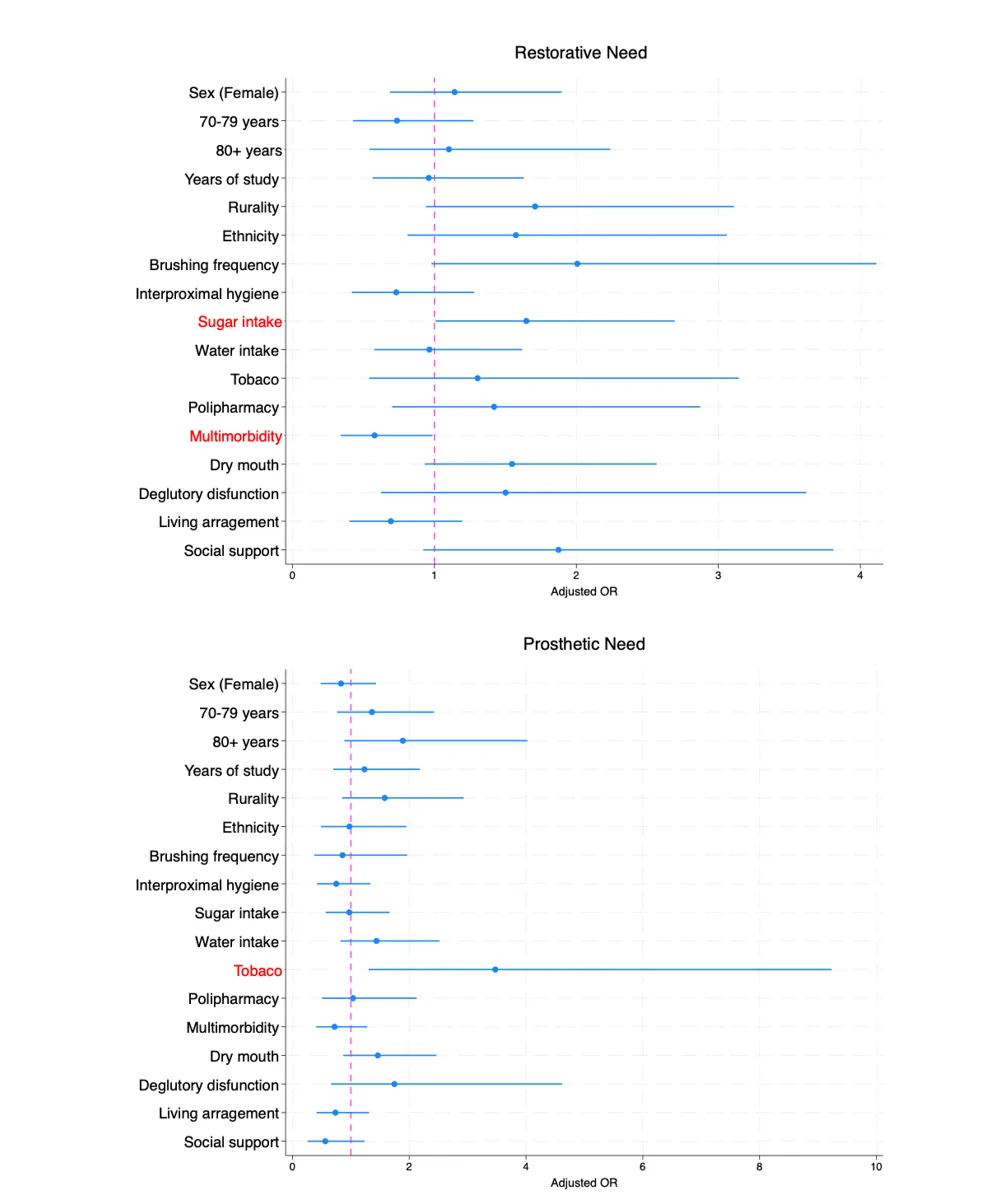
Adjusted associations between predictor variables and dental care needs. Forest plot showing adjusted odds ratios (aORs) and 95% CIs from separate multivariable logistic regression models for restorative treatment need and normative prosthetic treatment need. Points represent aORs, and horizontal bars represent 95% CIs. The vertical line indicates an odds ratio (OR) of 1 (no association). Statistically significant associations are highlighted in red.

## Discussion

### Principal Findings

This study highlights differences in both structural and intermediary social determinants of health between Indigenous (Mapuche) and non-Indigenous older adults in a sample from the La Araucanía Region of Chile. Compared with non-Indigenous participants, Mapuche individuals had lower educational attainment and were more likely to reside in rural areas. They also showed a higher prevalence of oral diseases, alongside a higher number of risk factors, including less frequent toothbrushing and higher sugar consumption. In addition, they had greater restorative treatment needs and reduced access to complex dental care services.

When examining the independent influence of structural and intermediary determinants on various oral health outcomes, structural factors showed more consistent associations than intermediary ones, in line with the WHO-CSDH framework. The associations observed for these variables may be consistent with the cumulative influence of structural disadvantage across the life course, whereby intermediary determinants in older age could reflect the long-term manifestation of social inequalities. Although our cross-sectional design does not allow direct assessment of life-course processes, longitudinal cohort research has demonstrated that early socioeconomic conditions shape oral health beliefs and behaviors, which subsequently influence adult clinical outcomes and quality of life, supporting the plausibility of cumulative pathways [73].

Notably, the pattern of associations varied depending on the outcome assessed. For instance, female sex was significantly associated with a higher prevalence of edentulism while showing lower odds of periodontitis. Similarly, lower educational attainment was associated with increased prevalence of nonfunctional dentition and edentulism; however, it showed no significant association with caries or periodontitis. For these latter outcomes, ethnicity showed the most consistent association. In particular, Mapuche participants had more than twice the odds of periodontitis compared with non-Mapuche counterparts (OR 2.43, 95% CI 1.21-4.88; *P*=.01). These findings are in line with international evidence reporting a 35% higher prevalence of periodontitis among Indigenous populations compared with non-Indigenous groups [2].

Regarding caries, Chilean adults aged 65 to 74 years exhibit one of the highest burdens worldwide (57,729 per 100,000) [74]. In our sample, all participants had a history of caries experience, and Mapuche participants showed a significantly higher prevalence of untreated (cavitated) caries compared with their non-Indigenous peers. This association remained significant in the multivariable model.

When examining variables related to access to dental care, no significant differences were observed in the frequency of the last dental visit between ethnic groups. However, the lower prevalence of complex treatments, such as dental implants and fixed prosthetic rehabilitations (eg, crowns and bridges), along with higher restorative treatment needs, including active caries and defective restorations, among Indigenous participants suggests a potential disparity in the quality of dental care received.

Ethnicity was significantly associated with 2 of the 9 outcomes in the multivariable models, a pattern similar to that observed for education, sex, and age. Within the WHO-CSDH framework, ethnicity is conceptualized as a structural determinant that interacts with socioeconomic, territorial, and cultural conditions, and is linked to cumulative disadvantages in health [6,9,75]. Historical and structural inequities have been documented as shaping access to services, use of dental prostheses, and the persistence of restorative treatment needs [4,5,76]. These findings highlight that ethnicity, alongside sex, age, and educational attainment, functions as a key axis of social stratification associated with differences in disease distribution and patterns of access and quality of treatment, rather than merely representing background characteristics.

Structural determinants are understood to shape differential exposure to intermediary determinants, including behavioral and biological risk factors [6,75]. In our study, unhealthy lifestyle behaviors, particularly the consumption of sugary snacks between meals, were more prevalent among Mapuche participants and were also linked to periodontitis and restorative treatment needs. These patterns are consistent with broader dietary changes reported in Indigenous populations, where colonization and globalization have contributed to the loss of traditional food systems, with a shift from subsistence diets toward store-bought and processed foods rich in refined sugars [77]. Structural factors, including corporate determinants of health such as the marketing of sugary products, may further reinforce this trend [78]. These dietary changes have been consistently linked with poorer oral health outcomes, including caries and periodontal disease [77]. The higher prevalence of periodontitis and caries in Mapuche participants may be related to a combination of factors, including ethnicity, dietary practices, and a lower brushing frequency, even though brushing frequency was not a significant predictor in the multivariable model. This finding supports the WHO-CSDH framework, which emphasizes the complex and not yet fully understood pathways through which structural determinants translate into biological outcomes [75].

Intermediary determinants played a more limited but still relevant role. Adverse outcomes were associated with dental visits occurring more than 2 years ago, frequent consumption of sugary snacks, and reduced toothbrushing frequency, while dry mouth, a biological factor linked with polypharmacy and aging [79], was also linked to both caries and nonfunctional dentition. Interestingly, better oral hygiene practices were associated with more recent dental visits. Addressing this requires expanding preventive interventions, particularly dietary counseling and management of xerostomia, as well as integrating tailored oral health education at the time of prosthetic delivery to improve long-term outcomes and avoid premature failures.

The Chilean health system, organized under a mixed public-private model, provides coverage to approximately 80% of the national population through its main government-funded scheme, FONASA, which primarily focuses on essential curative oral health care [3-5,80]. In this study, health insurance type was not included as a covariate since most participants were enrolled in FONASA, resulting in limited variability in coverage. Nevertheless, despite formal insurance coverage, a high burden of oral health problems alongside important gaps in service provision was observed. Although dental visit rates within the past 2 years were comparable across groups, 54% (206/383) of participants required restorative treatment, and a substantial proportion of existing restorations were found to be defective. Access to prosthetic care also showed notable gaps. While the national health guarantee (Explicit Health Guarantees [GES]) provides removable dentures for individuals at age 60 years, and other programs extend this benefit to vulnerable adults aged older than 20 years, important unmet needs persist [65,81-84]. In this study, 2 prosthetic indicators were considered. Unmet prosthetic need referred to individuals requiring a prosthesis but not currently wearing one, serving as a proxy for limited previous access to prosthetic care. In contrast, prosthetic treatment needs included both individuals requiring a new prosthesis and those with existing prostheses requiring repair or replacement, reflecting the overall demand for prosthetic treatment and providing an indicator relevant for service planning. Nearly one-fifth of participants presented unmet denture needs, and among those who already had dentures, almost 80% required repair or replacement. These findings underscore the importance of ensuring the quality of removable dentures provided in both public and private settings, particularly within publicly financed dental care, where more than 300,000 dentures are delivered annually in Chile [65]. A considerable proportion of these prostheses remain unused by patients due to poor fit, discomfort, or insufficient follow-up and adaptation protocols, which are critical to achieving functional rehabilitation and patient adherence [65]. The limited provision of complex treatments, coupled with gender-, education-, and ethnicity-based disparities in the use of fixed prostheses, is indicative of persistent inequalities. Within our sample, access to oral rehabilitation and preventive care appears more limited among older adults characterized by structural disadvantages, including advanced age, lower educational attainment, rural residence, and Indigenous ethnicity. Taken together, these patterns suggest that insurance coverage alone does not guarantee equity in oral health.

An innovative aspect of this study was the implementation of a teledentistry ecosystem (TEGO) supported by a mobile clinic. Although TEGO was designed for the broader older population, its pilot implementation in a Mapuche community demonstrated feasibility, user satisfaction, and a culturally respectful approach through the incorporation of intercultural facilitators, suggesting potential for supporting access to care in underserved communities [29]. Within the WHO-CSDH framework, strategies to reduce health inequities must address both structural and intermediary determinants of health [6,7]. In this context, technology-supported models of care may help mitigate structural barriers related to geographic isolation and limited service availability through remote consultation, screening, and triage, particularly in rural and underserved populations [44,85-88]. At the same time, digital health interventions may also influence intermediary determinants by strengthening oral health literacy, preventive behaviors, and patient engagement [85,89]. In this regard, the TEGO model illustrates a potentially equity-oriented strategy that combines digital tools with mobile service delivery to address barriers across the continuum of care. By facilitating access to primary oral health services (eg, restorative care and repair of removable prostheses) and, in some cases, enabling referral to secondary care for complex treatments such as implants, this approach may help reduce gaps in service use among populations facing structural disadvantages. Such strategies are particularly relevant for older adults living in rural or historically underserved settings and align with broader calls to action aimed at reducing health inequities and expanding access to care, including the objectives of United Nations Sustainable Development Goals, particularly goal 3 (good health and well-being) and goal 10 (reduced inequalities), while also contributing to the growing agenda of digital health equity [35,90-94].

The educational and ethnic inequalities observed in oral disease are consistent with prior evidence and support the need to account for differential disease burden in the design of public health policies [30,67,95]. Ongoing monitoring of these indicators may help identify service gaps and inform more equitable interventions. Even though a “one-size-fits-all” approach focused on individual treatment has done little to improve oral health outcomes at the population level, priority actions should focus on reducing structural disadvantages that influence access to and the cost of dental services, with a strong emphasis on equity [78]. This may include prioritizing socially disadvantaged groups and ensuring that treatments, such as dentures, are provided appropriately to individuals whose anatomical and functional characteristics allow effective use, while simultaneously establishing a subsidized system to improve access to more complex dental treatments for those who require them. Efforts should focus on strengthening the quality and follow-up of dental care, as well as implementing culturally tailored health promotion strategies for Indigenous and rural populations. Additionally, community-based delivery models, including mobile and digitally supported services, may contribute to reducing geographic and social inequities in access to care.

### Limitations and Generalizability

Certain limitations should be considered when interpreting these findings. First, the cross-sectional design precludes causal inference. The observed associations reflect patterns of co-occurrence rather than directional relationships, and reverse causation remains plausible. For example, poorer oral health may influence psychosocial domains such as depressive symptoms or social participation in older adults [96]. Accordingly, findings should be interpreted as evidence of social patterning rather than causal pathways.

Second, the sampling strategy and implementation context may have introduced selection bias and limited external validity. Participants were community-dwelling, functionally independent older adults recruited from a publicly funded, mobile, digitally supported care program implemented in municipalities selected for logistical feasibility. Participation required the capacity and willingness to attend scheduled dental visits, which likely underrepresents individuals with greater dependency, mobility limitations, or who are harder to reach—groups that may occupy more disadvantaged positions within the social gradient. This may have resulted in a healthier or more health-engaged sample, potentially underestimating the magnitude of oral health inequalities. At the same time, as an access-oriented intervention, the program may have attracted individuals with heterogeneous levels of perceived need, including both those seeking care for existing conditions and those engaging in preventive checkups, which may further influence the composition of the analytic sample.

Third, measurement limitations may have affected internal validity. Several intermediary determinants were self-reported, including dental visits, oral hygiene practices, current tobacco use, and chronic conditions used to define multimorbidity. These measures are susceptible to recall and social desirability bias, and the absence of detailed exposure assessment (eg, pack-years of smoking) or verification through clinical records may have introduced measurement error. Clinical outcomes were assessed by trained examiners using standardized protocols and direct supervision; however, periodontal and prosthetic assessments were more susceptible to field variability than caries, which was formally calibrated. These factors may have affected the precision and consistency of associations, particularly for periodontitis and for behavioral and biological variables.

Fourth, residual confounding cannot be excluded. Although the multivariable models were adjusted for the number of natural teeth as a key clinical factor, other potentially relevant confounders may not have been fully captured. In particular, the dataset lacked more granular measures of socioeconomic position (eg, wealth and income) and detailed health system characteristics, limiting the full operationalization of the structural dimension of the WHO-CSDH framework.

Fifth, missing data may have introduced bias. Although missingness was generally low, it was higher for periodontitis and polypharmacy. Incomplete periodontal examinations may occur under field conditions when full assessment is not feasible (eg, due to pain, tooth mobility, or time constraints during clinical evaluation). Missingness was not consistently associated with most measured characteristics, including sex, education, ethnicity, and rurality. However, for periodontitis, excluded participants were older than those included in the analysis, suggesting that examination feasibility may have differed by age. Given the overall low level of missingness and the limited evidence of systematic differences across most covariates, complete-case analysis was considered appropriate. Nonetheless, some degree of bias cannot be entirely excluded.

Finally, the absence of an a priori sample size calculation and the low frequency of some outcomes (eg, implants or complex rehabilitations) resulted in limited statistical precision for certain estimates, as reflected in wide CIs. Also, the study may not have been specifically powered to detect all potential differences between Mapuche and non-Mapuche participants; therefore, subgroup analyses should be interpreted cautiously.

Taken together, these findings are primarily applicable to community-dwelling, functionally independent older adults in rural and urban municipalities of La Araucanía, including both Mapuche and non-Indigenous individuals. Extrapolation to populations with higher levels of dependency or barriers to access, other regions of Chile, larger metropolitan areas, or Indigenous populations with different social or health care contexts should be undertaken cautiously. Moreover, as data were collected within a mobile, digitally supported care model specific to this program, generalizability to older adults receiving standard primary care services may be limited. This program-based implementation reflects real-world conditions of service delivery within its operational context, which may support ecological validity in terms of care processes and service pathways.

### Implications and Future Directions

Despite these limitations, this study contributes to the literature by examining oral health outcomes among older adults through a structured social determinants framework that simultaneously models structural determinants, including ethnicity, and intermediary factors. Ethnicity was associated only with cavitated caries and periodontitis. This analytical approach allows observed ethnic differences to be interpreted within broader social and territorial conditions, rather than attributing disparities to ethnic group membership alone. It does not eliminate ethnic gradients but situates them within patterned social conditions that may shape exposure, risk, and access over time [9]. In the Chilean context, national health surveys include self-identified Indigenous affiliation and have examined several social determinants, such as education, income, and insurance status. However, oral health outcomes have rarely been analyzed using multivariable frameworks that incorporate ethnicity alongside other determinants.

The findings further indicate that oral health inequalities in older adults may operate through multiple and outcome-specific pathways. Structural determinants—particularly age and educational attainment—were strongly associated with cumulative indicators of tooth loss, such as nonfunctional dentition and complete edentulism. In contrast, behavioral factors showed closer associations with selected access to dental treatment and treatment needs. This pattern suggests that different levels of determination may be more relevant depending on whether the outcome reflects long-term accumulation of disadvantage (eg, tooth loss) or more proximal conditions and service use patterns.

The inclusion of both rural and urban participants highlights the contextual dimension of these inequalities. While rural residence was not consistently associated with all clinical outcomes, it was linked to selected treatment-related indicators, suggesting that territorial factors may influence patterns of rehabilitation and unmet need even when not uniformly affecting disease prevalence.

Future longitudinal studies incorporating more detailed measures of lifetime exposures, including behavioral factors such as smoking history, as well as health service trajectories and contextual factors, would help clarify the pathways linking structural determinants to oral health outcomes in later life. Research may also benefit from intersectional approaches capable of examining how structural determinants interact to shape oral health disadvantages. Additionally, evaluating the long-term effectiveness and equity of innovative service delivery models, such as teledentistry and mobile dental clinics, may provide valuable evidence to inform strategies aimed at reducing disparities. Mixed methods studies incorporating qualitative perspectives on social, cultural, and system-level barriers may further contribute to a more comprehensive understanding of the mechanisms underlying the observed patterns.

### Conclusions

Oral health inequalities differed between Mapuche and non-Indigenous older adults in La Araucanía and were associated with both structural and behavioral determinants. Structural determinants, particularly lower education, female sex, older age, and ethnicity, showed consistent associations with oral health status, whereas dental care access reflected a combination of structural and behavioral factors. In contrast, current treatment needs were mainly associated with behavioral factors, including sugar consumption and tobacco use. Although Mapuche participants exhibited poorer oral health, higher restorative needs, and reduced access to complex dental care in bivariate analyses, ethnicity remained associated only with cavitated caries and periodontitis in adjusted models.

These findings underscore the importance of incorporating social determinants into oral health planning for underserved older adults, particularly in Indigenous and rural populations experiencing persistent social and territorial inequities. By applying the WHO-CSDH framework within a hybrid teledentistry and mobile clinic model, this study illustrates how digitally supported territorial care programs can generate clinically grounded population health data that help identify inequities in oral health status, access to care, and treatment needs. Integrating these approaches into oral health information systems and service planning may support more equity-oriented prioritization, resource allocation, and delivery of preventive, restorative, and prosthetic care for older adults in underserved territories.

## Data Availability

The datasets analyzed during this study are not publicly available due to privacy restrictions regarding the Indigenous population but are available from the corresponding author on reasonable request, subject to ethical approval.

## Appendix

Multimedia Appendix 1 Multivariable logistic regression of structural and intermediary determinants on access to dental care–related outcomes.

Multimedia Appendix 2 Multivariable logistic regression of structural and intermediary determinants on dental treatment needs.

## Abbreviations

CAL: clinical attachment level
CAPREDENA: National Defense Social Security Fund
CSDH: Commission on Social Determinants of Health
DIPRECA: Carabineros de Chile Social Security Directorate
DMFT: decayed, missing, and filled teeth
EAT-10: Eating Assessment Tool-10
FONASA: National Health Fund
GES: Explicit Health Guarantees
ICDAS: International Caries Detection and Assessment System
ISAPRE: private health insurance institution
NHANES III: Third National Health and Nutrition Examination Survey
OR: odds ratio
PD: probing depth
PRAIS: Comprehensive Health Care and Reparation Program for Victims of Human Rights Violations
RSH: Social Registry of Households
TEGO: Geriatric Dental Specialties Tele-platform
WHO: World Health Organization
