# Supplementary material for: Social Determinants and Oral Health Among Indigenous and Non-Indigenous Older Adults in La Araucanía, Chile: Cross-Sectional Study
Source: JMIR Aging. 2026 Sep 29;9:e85075. doi: 10.2196/85075 (PMC13662451; doi:10.2196/85075)
Supplement: Supplementary file 1 [file aging_v9i1e85075_app1.pdf]

Multimedia Appendix 1

Table. Multivariable logistic regression of structural and intermediary determinants on access to dental care related outcomes

Variable	Categories	Fixed Dental Prostheses[a]		Unmet denture need[b]		Last dental visit (≤ 2 years)[c]	
		OR	*P value*	OR	*P value*	OR	*P value*
**Structural Determinants**							
Sex[b]	Male	1.00 (ref)		1.00 (ref)		1.00 (ref)	
	Female	3.46	**.003**	0.69	.27	0.77	.24
Age Groups	60-69	1.00 (ref)		1.00 (ref)		1.00 (ref)	
	70-79	2.04	.10	1.04	.91	0.95	.84
	≥80	5.05	**.004**	0.87	.77	0.76	.38
Educational attainment[a,c]	>8 years	1.00 (ref)		1.00 (ref)		1.00(ref)	
	≤8 years	0.37	**.03**	1.73	.14	0.67	.09
Rurality[a]	No rural	1.00 (ref)		1.00 (ref)		1.00 (ref)	
	Rural	1.38	.5	2.86	**.008**	1.11	.68
Ethnicity[a]	None	1.00 (ref)		1.00 (ref)		1.00(ref)	
	Mapuche	0.49	.2	0.44	.07	0.83	.51
**Intermediary Determinants**							
**a) Behavioral factors**							
Daily Brushing Frequency[b,c]	0-1	1.00 (ref)		1.00 (ref)		1.00 (ref)	
	≥2	0.46	.20	0.25	**<.001**	1.92	**.04**
Interproximal oral hygiene[a,c]	Yes	1.00 (ref)		1.00 (ref)		1.00 (ref)	
	No	1.84	.12	0.91	.82	1.90	**.01**
Sugary snacks consumption[a]	No	1.00 (ref)		1.00 (ref)		1.00 (ref)	
	Yes	3.48	**.002**	0.62	.17	1.34	.18
Daily water intake (≥2lts)	Yes	1.00 (ref)		1.00 (ref)		1.00 (ref)	
	No	1.4	.42	1.64	.16	1.12	.63
Smoking habit	Non-smoker	1.00 (ref)		1.00 (ref)		1.00 (ref)	
	Smoker	1.09	.88	1.19	.75	1.49	.29
**b) Biological factors**							
Polypharmacy	No	1.00 (ref)		1.00 (ref)		1.00 (ref)	
	Yes	1.86	.20	1.76	.21	1.52	.16
Multimorbidity	No	1.00 (ref)		1.00 (ref)		1.00 (ref)	
	Yes	0.96	.92	0.57	.13	1.45	.12
Dry mouth[a]	No	1.00 (ref)		1.00 (ref)		1.00 (ref)	

	Yes	0.62	.24	1.70	.13	0.86	.51
Swallowing disorders	No	1.00 (ref)		1.00 (ref)		1.00 (ref)	
	Yes	0.60	.47	0.69	.54	0.90	.78
**c) Psychosocial factors**							
Living arrangement	With family	1.00 (ref)		1.00 (ref)		1.00 (ref)	
	Alone	1.11	.79	1.17	.66	0.78	.59
Social support	Yes	1.00 (ref)		1.00 (ref)		1.00 (ref)	
	No	1.17	.80	0.74	.50	0.84	.06

Note: For each outcome, an independent multivariable logistic regression model was fitted including all predictors and the number of teeth. a) Significant bivariate association with Fixed Dental Prostheses. b) Significant bivariate association with Unmet denture need. c) Significant bivariate association with Last dental visit.
