# Supplementary material for: Social Determinants and Oral Health Among Indigenous and Non-Indigenous Older Adults in La Araucanía, Chile: Cross-Sectional Study
Source: JMIR Aging. 2026 Sep 29;9:e85075. doi: 10.2196/85075 (PMC13662451; doi:10.2196/85075)
Supplement: Supplementary file 2 [file aging_v9i1e85075_app2.pdf]

Multimedia Appendix 2

Table. Multivariable logistic regression of structural and intermediary determinants on dental treatment needs

Variable	Categories	Restorative treatment need[a] OR	Restorative treatment need[a] *P value*	Prosthetic treatment needs[b] OR	Prosthetic treatment needs[b] *P value*
**Structural Determinants**					
Sex	Male	1.00 (ref)		1.00 (ref)	
	Female	1.15	.58	0.85	.57
Age Group [b]	60-69	1.00 (ref)		1.00 (ref)	
	70-79	0.73	.26	1.35	.31
	≥80	1.10	.89	1.79	.13
Educational attainment[b]	>8 years	1.00 (ref)		1.00 (ref)	
	≤8 years	0.96	.89	1.28	.39
Rurality[a, b]	No rural	1.00 (ref)		1.00 (ref)	
	Rural	1.72	.07	1.58	.14
Ethnicity[a]	None	1.00 (ref)		1.00 (ref)	
	Mapuche	1.56	.19	0.98	.96
**Intermediary Determinants**					
**a) Behavioral factors**					
Daily Brushing Frequency[a, b]	0-1	1.00 (ref)		1.00 (ref)	
	≥2	2.01	.59	0.82	.65
Interproximal oral hygiene[b]	Yes	1.00 (ref)		1.00 (ref)	
	No	0.75	.32	0.74	.31
Sugary snacks consumption[a]	No	1.00 (ref)		1.00 (ref)	
	Yes	1.69	**.03**	0.98	.95
Daily water intake (≥2lts)	Yes	1.00 (ref)		1.00 (ref)	
	No	0.97	.91	1.45	.18
Smoking habit	Non-smoker	1.00 (ref)		1.00 (ref)	
	Smoker	1.34	.51	3.48	**.01**
**b) Biological factors**					
Polypharmacy	No	1.00 (ref)		1.00 (ref)	
	Yes	1.44	.30	1.02	.95
Multimorbidity[a]	No	1.00 (ref)		1.00 (ref)	
	Yes	0.59	.05	0.73	.28
Dry mouth[b]	No	1.00 (ref)		1.00 (ref)	

	Yes	1.55	.08	1.48	.14
Swallowing disorders	No	1.00 (ref)		1.00 (ref)	
	Yes	1.51	.35	1.75	.25
**c) Psychosocial factors**					
Living arrangement	With family	1.00 (ref)		1.00 (ref)	
	Alone	0.67	.16	0.74	.31
Social support[a]	Yes	1.00 (ref)		1.00 (ref)	
	No	1.85	.08	0.57	.16
**d) Health System factors**					
Self-reported time since last dental visit[b]	>2 years	1.00 (ref)		1.00 (ref)	
	≤ 2 years	0.89	.64	1.25	.39

Note: For each outcome, an independent multivariable logistic regression model was fitted including all predictors and the number of teeth. a) Significant bivariate association with Restorative treatment need. b) Significant bivariate association with Prosthetic treatment needs.
